# *Colletotrichum* Species Associated with Japanese Plum (*Prunus salicina*) Anthracnose in South Korea

**DOI:** 10.1038/s41598-019-48108-1

**Published:** 2019-08-19

**Authors:** Oliul Hassan, Yong Se Lee, Taehyun Chang

**Affiliations:** 1 Department of Ecology & Environmental System, College of Ecology & Environmental Sciences, Kyungpook National University, Sangju, Gyeongbuk 37224 Republic of Korea; 2 Division of Life and Environmental Sciences, College of Life and Environmental Sciences, Daegu University, Gyeongsan, Gyeongbuk 38453 Republic of Korea

**Keywords:** Fungal pathogenesis, Pathogens

## Abstract

A total of 24 *Colletotrichum* isolates were isolated from diseased Japanese plum (*Prunus salicina*) fruits showing chlorotic regions with whitish-brown sunken necrotic lesions and phylogenetic relationships among the collected *Colletotrichum* isolates were determined. A subset of 11 isolates was chosen for further taxonomic study based on morphology and molecular characteristics identified using the internal transcribed spacer (ITS) and beta-tubulin (TUB2) genes. Isolates in the *C*. *acutatum* complex were analyzed using partial sequencing of five gene regions (ITS, GAPDH, ACT, TUB2, and CHS), and *C*. *gloeosporioides* sensu lato (s.l.) isolates were analyzed using seven gene regions (ITS, TUB2, GAPDH, ACT, CAL, CHS-1, and ApMat). Morphological assessments in combination with phylogenetic analysis delineated four species of *Colletotrichum* including *C*. *gloeosporioides* sensu stricto (s.s.), *C*. *nymphaeae*, *C*. *foriniae*, and *C*. *siamense*; these data identify *Colletotrichum fioriniae* and *C. siamense* two new species associated with plum anthracnose in South Korea. Finally, the pathogenicity of these four species in the development of plum anthracnose in South Korea was confirmed by inoculations of plum fruit.

## Introduction

Japanese plums (*Prunus salicina* Lindl.) are delicious stone fruits, which have a wide variety of uses. Consumers typically prefer to eat fresh Japanese plums for their characteristic taste, though a small percentage prefer them dry. They can also be used in jams or jellies. The fruits are rich in carbohydrates (sucrose, glucose, and fructose), malic acid, phenolic compounds (chlorogenic acid, neochlorogenic acid), anthocyanins (cyanidin-3-glucoside, cyaniding-3-rutinoside), vitamin C, β-carotene, and minerals (potassium, phosphorus)^1^. Though native to China the name Japanese plum derives from the fruit tree first being imported into the USA from Japan^2^. Japanese plums are cultivated along with apples, peaches, oranges, and Asian pears in South Korea. Both the cultivation area and production of Japanese plums in South Korea increased from 2007 (5,803 ha, 64,816 tons) to 2015 (5,920 ha, 67,810 tons)^3^. The production of Japanese plum fruits in Korea can be negatively impacted by various factors including different diseases including fungal diseases (brown rot, gray mold, leaf spot, plum pocket, and powdery mildew) and bacterial diseases (bacterial black spot, shot hole, etc.)^4–8^. Recently anthracnose of Japanese plum caused by *Colletotrichum* species has been reported in Korea^3,9^.

Most *Colletotrichum* species are plurivorous anthracnose pathogens that cause disease in a wide range of hosts, including fruit trees and vegetables^10,11^. The most characteristic symptom enabling the recognition of anthracnose is the presence of sunken necrotic lesions on leaves, stems, flowers, and fruit, which limits the quality of agricultural products (fruits, flowers). *Colletotrichum* species have also been reported to caused anthracnose in common fruits in Korea, such as apples, grapes, peaches, and persimmons^12–15^. Multiple *Colletotrichum* species can infect a single fruit cultivar. In Korea, *Colletotrichum acutatum* and *C*. *gloeosporioides* sensu stricto (s.s.) are responsible for bitter rot of apples and anthracnose of peaches; *C*. *acutatum*, *C*. *gloeosporioides* s.s., and *C. viniferum* for ripe rot of grapes; and *C*. *acutatum*, *C*. *gloeosporioides* s.s., *C*. *horii*, and *C*. *siamense* for anthracnose of persimmons^12–14,16,17^. To date, *C*. *acutatum, C*. *gloeosporioides* s.s., and *C*. *nymphaeae* have been reported as the causal agents of plum anthracnose in Korea^3,9^. Lee *et al*. identified C. *acutatum* and *C*. *gloeosporioides* s.s. (causal agents of plum anthracnose) based on morphology and internal transcribed spacers (ITS) sequence data^9^. Methods for identifying *Colletotrichum* species based on morphology and ITS sequences are not reliable for species discrimination within *Colletotrichum*, although they can be helpful in the resolution of species complexes or clades^18–20^. A recently developed multi-locus sequence analysis approach combined with morphological evaluation revealed that *C*. *gloeosporioides* sensu lato (s.l.) and *C*. *acutatum* s.l., each comprise a species complex^20,21^. *C*. *gloeosporioides* s.s. is a strictly defined species ((*Colletotrichum gloeosporioides* (Penz.) Penz. & Sacc)) excluding other species within the C. *gloeosporioides* species complex, while *C*. *gloeosporioides* s.l. includes other *Colletotrichum* species of this complex^20^. In addition, the ApMat marker gene has been used to resolve and improve the systematic classification of *Colletotrichum* species complexes, such as *C*. *gloeosporioides* and *C*. *siamense* complexes^22–24^. The use of a five genes phylogenetic analyses along with morphological characters to identify *C*. *nymphaeae* as the causative agent of plum anthracnose revealed that *Colletotrichum* species associated with plum anthracnose in Korea may have a remarkable species diversity^9^.

Therefore, this study sought to investigate species diversity within *Colletotrichum* isolates related to plum anthracnose in Sangju, Korea based on combined morphological and multigene phylogenetic strategies, followed by a pathogenesis analysis of the different identified *Colletotrichum* species on plum fruit.

## Results

### Isolation and preliminary identification of *Colletotrichum* species

**Figure 1:**
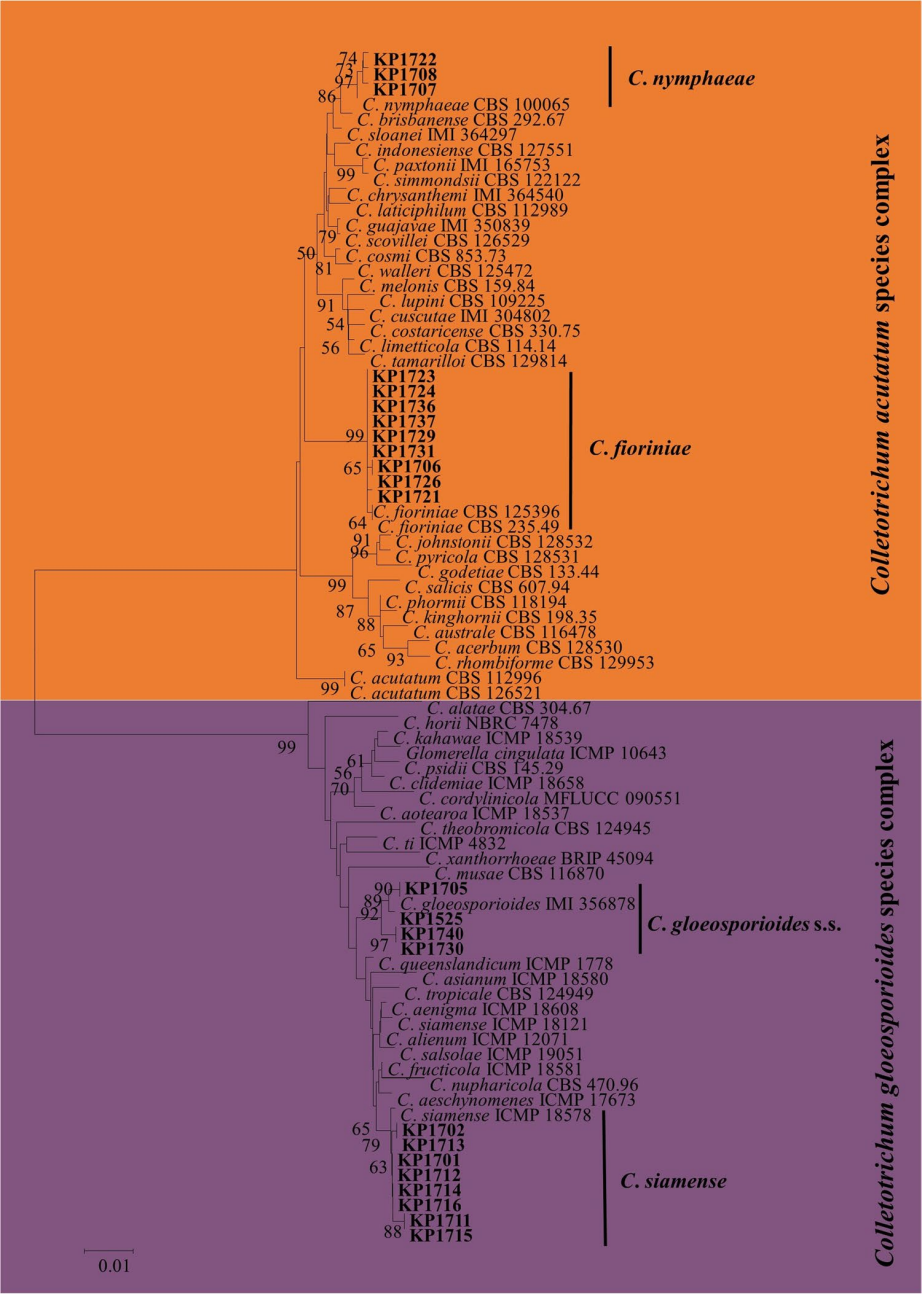
Neighbor-joining (NJ) tree derived from concatenated sequence alignment of ITS and TUB2 showing the separation of *Colletotrichum* isolates into the *C*. *acutatum* species complex and *C*. *gloeosporioides* s.l. (indicated by colored blocks). Bootstrap support values (ML > 50) are given at the nodes.

A total of 24 *Colletotrichum* isolates were isolated from Japanese plum fruits collected from different commercial orchards exhibiting anthracnose in Sangju, South Korea (Gyeongbuk Province). The *Colletotrichum* spp. were isolated and preliminarily identified based on colony and conidial morphology. Among the 24 isolates, 15 colonies were gray to white and produced subcylindrical to cylindrical conidia similar to that of *C*. *gloeosporioides* s.l.^20^. Colonies of the remaining nine isolates were pinkish in color and produced fusiform conidia, which is common of fungi in the *C. acutatum* species complex^21^. *Colletotrichum* isolates belonging to *C. gloeosporioides* s.l., (12 isolates) and *C. acutatum* s.l., (12 isolate) were first delineated using the combined ITS and TUB2 align sequence data set for phylogenetic analysis (Fig. 1). Six isolates of *C*. *gloeosporioides* s.l., (four from *C*. *siamense* clade and two from *C. gloeosporioides* s.s clade) and five isolates of *C. acutatum* s.l., (three from *C. fioriniae* clade and two from *C*. *nymphaeae* clade) identified based on ITS and TUB2 sequences data were selected for further phylogenetic analysis (Fig. 1).

### Phylogenetic analyses of the combined datasets

**Figure 2:**
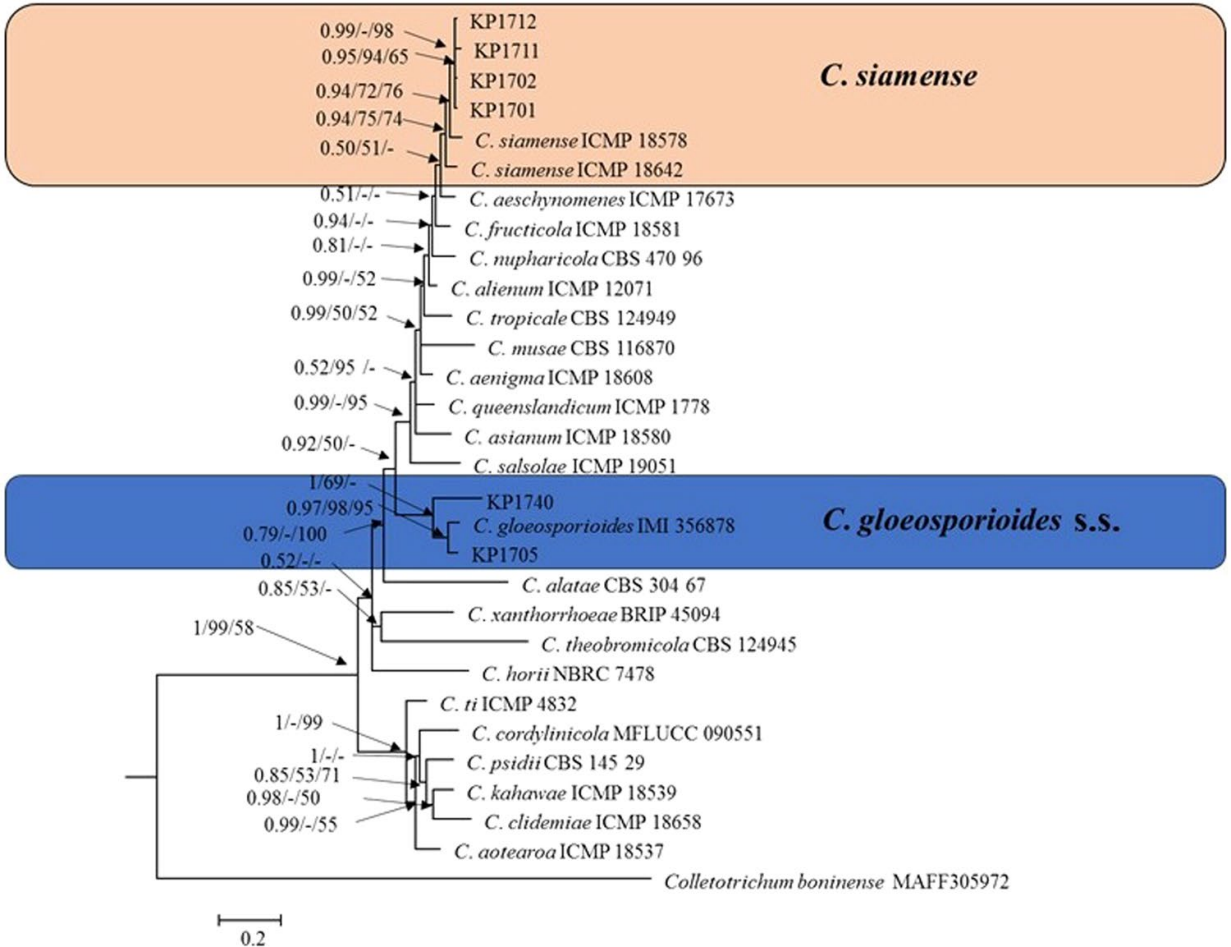
Bayesian phylogeny (BI) based on a 50% majority rule consensus tree using combined sequence alignment of ITS, TUB2, GAPDH, ACT, CAL, and CHS-1. Colored blocks indicate the two clades containing plum isolates. Bayesian posterior probability values ≥ 0.5 and bootstrap support values ≥ 50% of maximum parsimony analysis and maximum likelihood analysis are given at the nodes. The scale bar shows the number of substitutions expected per site. *Colletotrichum boninense* MAFF305972 was used as the out-group.

*Colletotrichum gloeosporioides* s.l. isolates were identified at the species level using a six-gene phylogenetic analysis (Fig. 2). Thirty sequences were present in the combined aligned data matrix (ITS, TUB2, GAPDH, ACT, CAL, CHS-1), which included *C. boninense* (CBS 123755) as the outgroup and 1,566 characters, as well as gaps in the alignment. The *C*. *gloeosporioides* species complex phylogram showed that isolates of the plum clustered in two clades (Fig. 2). Two isolates (KP1705 and KP1740) clustered with *C*. *gloeosporioides* s.s., ex-type isolate (IMI356878) with a high bootstrap support/posterior probability value (69%/1.00) and could be identified with confidence as *C*. *gloeosporioides* s.s. The remaining four isolates (KP1701, KP1702, KP1711 and KP1712) formed a sister clade with *C*. *siamense* ex-type isolates (ICMP I8578and ICMP I8642). The isolates KP1701, KP1702, KP1711 and KP1712 were further confirmed as *C*. *siamense* by phylogenetic analysis using ApMat sequences data (Appendix 1).

**Figure 3:**
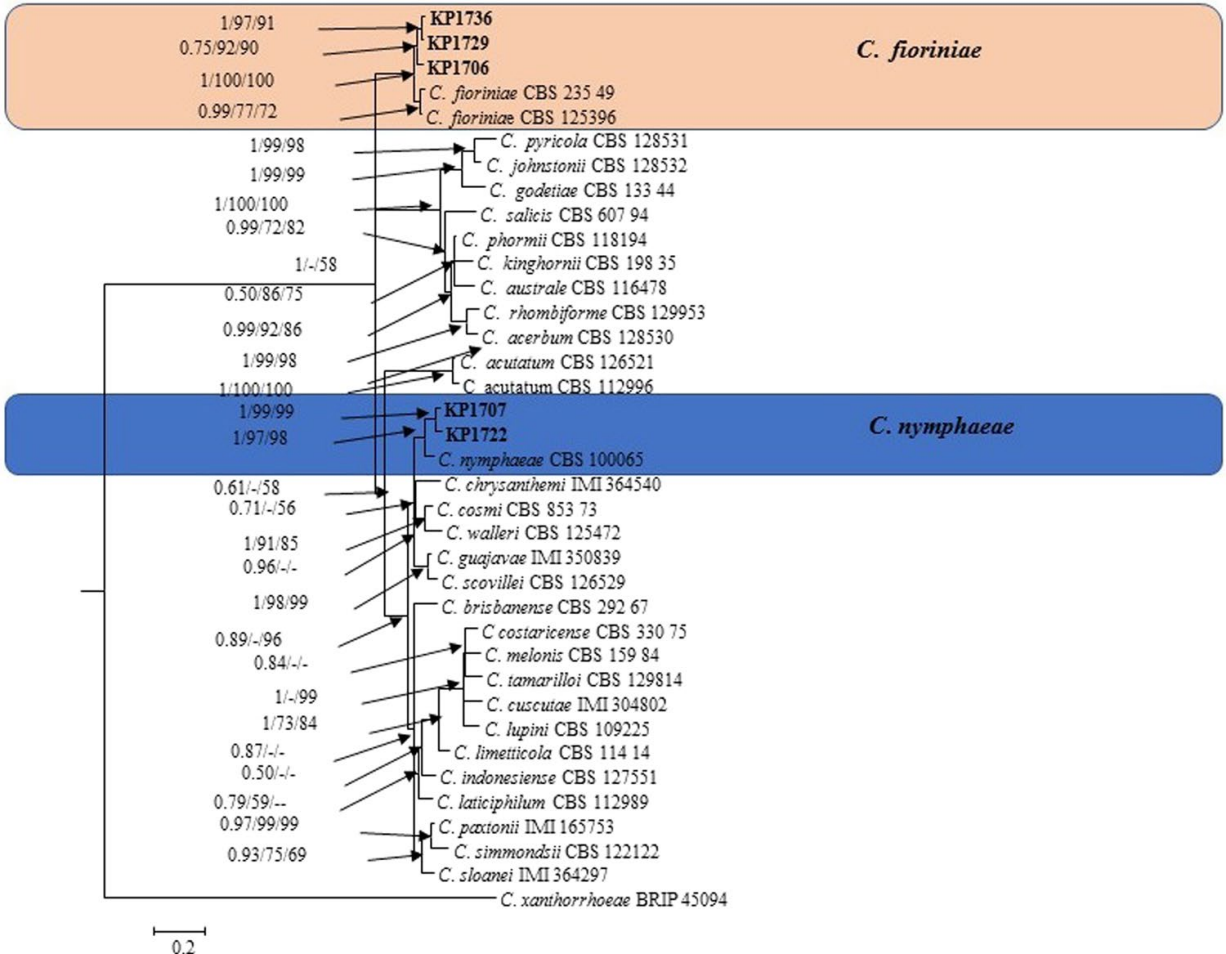
Bayesian phylogeny (BI) according to a 50% majority rule consensus tree using combined sequence alignment of ITS, TUB2, GAPDH, ACT, and CHS-1. Colored blocks indicate the two clades containing plum isolates. Bayesian posterior probability values ≥ 0.5 and bootstrap support values ≥ 50% of maximum parsimony analysis and maximum likelihood analysis are given at the nodes. The scale bar shows the number of substitutions expected per site. *C*. *xanthorrhoeae* BRIP 45094 was used as the out-group.

The phylogram in Fig. 3 shows the isolates identified in the *C. acutatum* species complex. The five-gene (ITS, TUB2, GAPDH, ACT, and CHS-1) phylogenetic analysis of *C*. *acutatum* s.l. contained 38 sequences, including the outgroup *C*. *xanthorrhoeae* (BRIP 45094). Three isolates (KP1706, KP1729, and KP1736) could be identified as *C. fioriniae* as it was in the same clade as *C. fioriniae* isolates CBS 23549 and CBS 125396 and showed robust posterior probability and bootstrap support values (1.00 and 100%) (Fig. 3). Two isolates (KP1707, and KP1722) clustered with the *C*. *nymphaeae* ex-type isolate CBS 100065 (bootstrap support/posterior probability value 97%/1.00) and were identified as *C*. *nymphaeae*.

### Pairwise homoplasy index (PHI) test

The concept of Genealogical Concordance Phylogenetic Species Recognition (GCPSR) was used to analyze phylogenetically related but ambiguous species. The pairwise homoplasy index (PHI) test found significant recombination between *C. siamense* and four closely related strains (KP1701, KP1702, KP1711 and KP1712) (Φw = <0.001), *C*. *gloeosporioides* s.s., and two closely related strains (KP1705and KP1740) (Φw = <0.003), *C*. *nymphaeae* and two closely related strains (KP1707 and KP1722) (Φw = <0.002), and *C*. *fioriniae* and three closely related strains (KP1706, KP1729, and KP1736) (Φw = <0.01) (Appendix 2).

### Taxonomy

***Colletotrichum siamense*** Prihastuti, L. Cai and K.D. Hyde, Fungal Diversity 39: 158. 2009 Fig. 4.

**Figure 4:**
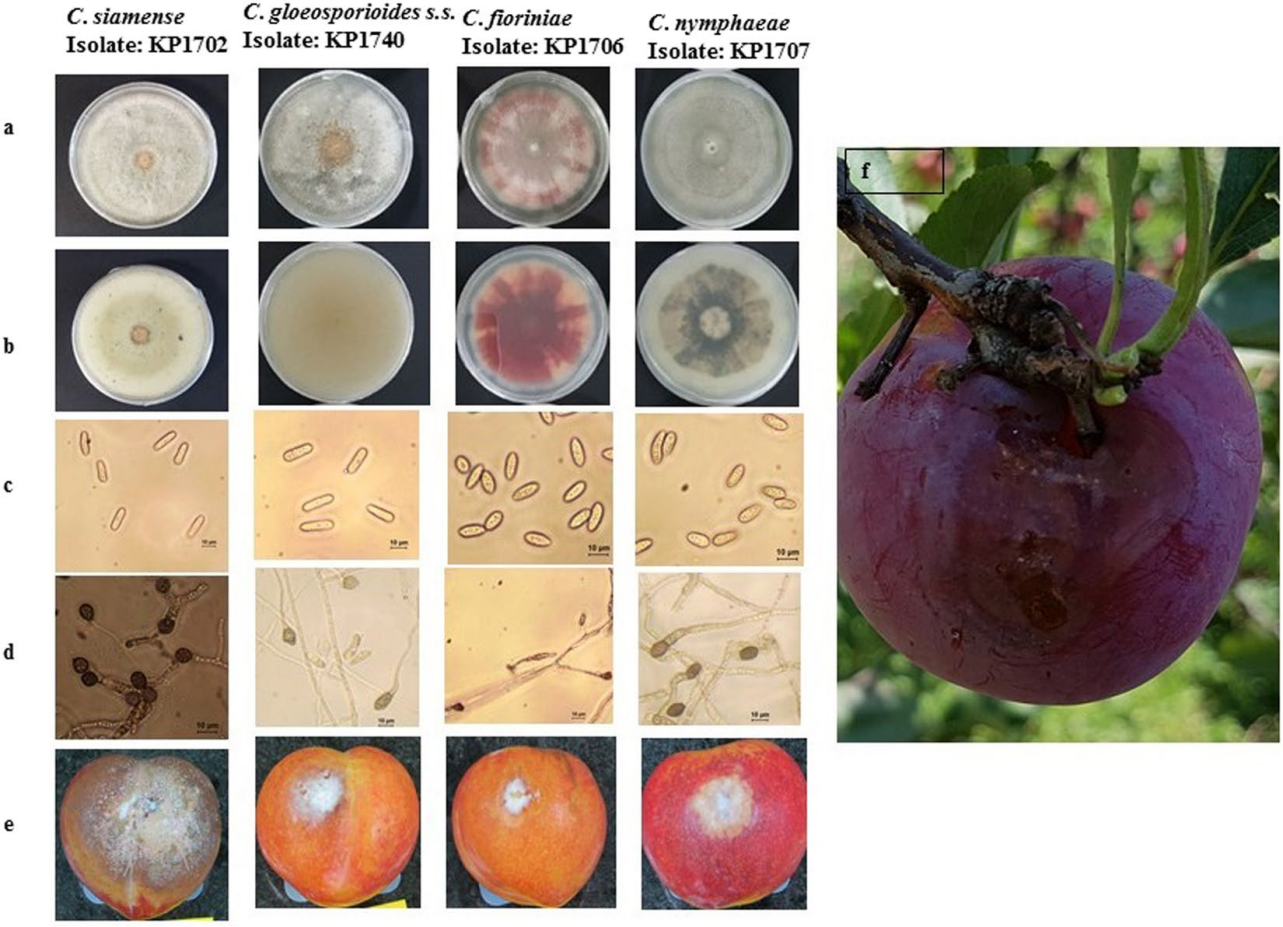
*C. siamense* (KP1702), *C. gloeosporioides* s.s., *(KP1740)*, *C. fioriniae (KP1706) and C*. *nymphaeae* (KP1707) (from left to right). (**a,b**) Colonies on PDA of different *Colletotrichum* species isolates (KP1702, KP1740, KP1706 and KP1707 from left to right). (**c**) Conidia of different isolates of *Colletotrichum* species isolates (KP1702, KP1740, KP1706 and KP1707 from left to right). (**d**) Appressoria of different isolates of *Colletotrichum* species isolates (KP1702, KP1740, KP1706 and KP1707 from left to right). (**e**) Symptoms of anthracnose on artificially inoculated plum fruits after 12 days of inoculation by the wounding method (KP1702, KP1740, KP1706 and KP1707 from left to right). (**f**) Symptoms of anthracnose on naturally infected plum fruits. Scale: (**c**,**d**) = 10 μm.

#### Description

*Colonies* on Difco potato dextrose agar (PDA) grew to 70–76 mm in diameter at a rate of 10.9 mm/day after seven days at 28 °C in the dark. Colonies were creamy white with aerial mycelium, and there were yellowish white masses of conidial ooze. The colonies were pale yellow in reverse. Conidia were hyaline, aseptate, smooth, cylindrical, straight or slightly curved, slightly tapered toward the end, 17.8–24.2 × 5.0–7.3 μm, av ± SD = 19.8 ± 1.7 × 6.3 ± 0.60, L/W ratio = 3.0, n = 50. Appressoria globose to ellipsoid, without lobes, dark brown, unbranched, 6.8–12.20 × 6.7–10.3 μm, av ± SD   8.70 ± 1.20 × 8.21 ± 0.80 μm, L/W ratio = 1.1, n = 50 (Table 1; Fig. 4).

**Table 1:** Morphological data of *Colletotrichum* isolates.

Taxon	Isolates	Colony morphology	Conidia morphology			Appressoria	morphology
			Length (average ± SD)	Width (average ± SD)	Shape	Length (average ± SD)	Width (average ± SD)
*C. siamense*	KP1701	Creamy white, formed a thin layer over the PDA with white aerial mycelium and conidia produced across the PDA plate	19.40 ± 1.10 μm	5.90 ± 0.44 μm	Cylindrical, straight to slight curve, not fusiform but rather slightly tapered toward the end	9.54 ± 1.12 μm	7.20 ± 0.54 μm
	KP1702	Creamy white, formed a thin layer over the PDA with white aerial mycelium and conidia produced across the PDA plate	19.81 ± 1.71 μm	6.32 ± 60 μm	Cylindrical, straight to slight curve, not fusiform but rather slightly tapered toward the end	8.70 ± 1.20 μm	8.21 ± 0.80 μm
	KP1711	Creamy white, formed a thin layer over the PDA with white aerial mycelium and conidia produced across the PDA plate	19.32 ± 1.23 μm	5.60 ± 0.60 μm	Cylindrical, straight to slight curve, not fusiform but rather slightly tapered toward the end	9.17 ± 1.41 μm	7.20 ± 0.74 μm
	KP1712	Creamy white, formed a thin layer over the PDA with white aerial mycelium and conidia produced across the PDA plate	19.41 ± 1.10 μm	7.03 ± 0.62 μm	Cylindrical, straight, obtuse end	9.81 ± 1.00 μm	7.31 ± 0.80 μm
*C. gloeosporioides* s.s.	KP1705	Dense cottony, pale orange	16.63 ± 1.74 μm	7.27 ± 0.52 μm	Cylindrical, round at both ends	9.60 ± 1.08 μm	8.08 ± 1.42 μm
	KP1740	Dense cottony, gray	17.90 ± 1.60 μm	5.93 ± 0.80 μm	Cylindrical, round at both ends	12.95 ± 1.60 μm	8.66 ± 1.00 μm
*C. fioriniae*	KP1706	Pink with white aerial mycelium	14.52 ± 1.01 μm	5.90 ± 0.71 μm	Fusiform	9.25 ± 1.02 μm	8.12 ± 0.52 μm
	KP1729	Pink with white aerial mycelium	14.62 ± 1.41 μm	5.61 ± 0.71 μm	Fusiform	11.08 ± 1.35 μm	7.26 ± 1.02 μm
	KP1736	Pink with white aerial mycelium	12.89 ± 1.35 μm	4.90 ± 0.84 μm	Fusiform	11.86 ± 1.78 μm	8.20 ± 1.04 μm
*C. nymphaeae*	KP1707	Gray with light aerial mycelium	10.37 ± 1.92 μm	4.32 ± 0.65 μm	Subcylindrical, round at both ends or slightly tapered at one end	9.83 ± 1.24 μm	7.71 ± 1.54 μm
	KP1722	Gray with light aerial mycelium	9.73 ± 1.51 μm	4.23 ± 0.64 μm	Subcylindrical, round at both ends or slightly tapered at one end	10.80 ± 1.78 μm	7.15 ± 1.05 μm

#### Materials examined

SOUTH KOREA, Gyeongbuk Province, Sangju City, from diseased fruit of *Prunus salicina*, 22 Jul. 2017, O. Hassan, culture KP1701, KP1702, KP1711 and KP1712.

#### Notes

*Colletotrichum siamense* has been identified as the causative agent of anthracnose of *Malus pumila* and *Diospyros kaki* in South Korea^17,25^. *Colletotrichum siamense* is believed to have first infect coffee berries in Thailand; it has been reported as a pathogen on various hosts and is now considered a biologically and geographically diverse species^17,20,26^. Based on multi-locus (ITS, TUB2, GAPDH, ACT, CAL, and CHS-1) phylogenetic analysis, KP1701, KP1702, KP1711 and KP1712 isolates were identified as *C*. *siamense* (Fig. 2). A phylogenetic tree based on ApMat sequences also revealed that *C*. *siamense* species formed different clades, and KP1701, KP1702, KP1711 and KP1712 clustered together with one *C*. *siamense* species clade (Appendix 1). This result is consistent with recent publications from Sharma *et al*.^23,24^. Although *C*. *siamense* species isolates clustered in different clades, they are considered a single species rather than a species complex^23,24,27^. The closest matches (99% identity) in a BLAST search using ApMat sequence of previously identified strains were YT02, SQ01, and LQ22 from China^28^.

*Colletotrichum gloeosporioides* (Penz.) Penz. & Sacc., Atti Reale Ist. Veneto Sci. Lett. Arti., Serie 6, 2: 670. 1884.

For detailed description of *C*. *gloeosporioides* s.s., see Cannon *et al*.^29^ and Weir *et al*.^20^.

#### Materials examined

SOUTH KOREA, Gyeongbuk Province, Sangju City, from diseased fruit of *Prunus salicina*, 20 Jul. 2017, O. Hassan, Culture KP1705 and KP1740 (Fig. 4).

Notes: *Colletotrichum gloeosporioides* s.s., was reported to be the causative agent of anthracnose on various host plants, including *Malus prunifolia*, *Ficus carica*, *Liriodendron chinense*, *Prunus avium*, and *Diospyros kaki* in South Korea^6,30–33^. Previously, *C*. *gloeosporioides* s.s. was isolated from *Prunus salicina* from Daegu area^3^. This species was isolated from *Prunus salicina* from the Sangju area in the present study. This study identified the isolates KP1705 and KP1740 as *C*. *gloeosporioides* s.s. based on morphology and multi-locus (ITS, TUB2, GAPDH, ACT, CAL, and CHS-1) phylogenetic analysis. In the phylogram, these isolates clustered in the same clade with *C. gloeosporioides* s.s. (IMI 356878) with 70% bootstrap support and posterior probability value of 1.00 (Fig. 2).

***Colletotrichum fioriniae*** (Marcelino & Gouli) R.G. Shivas & Y.P. Tan, Fungal Diversity 39: 117. 2009. Description and illustrations: see Damm *et al*.^21^.

Materials examined: SOUTH KOREA, Gyeongbuk Province, Sangju City, from diseased fruit of *Prunus salicina*, 21 Jul. 2017, O. Hassan, Culture KP1706, KP1729 and KP1736 (Fig. 4).

Notes: *Colletotrichum fioriniae* has been reported as the causative agent of anthracnose on various host plants, including *Lycium chinense* and *Solanum melongena* in Korea^6,14,34^. In this study, *C*. *fioriniae* was isolated from *Prunus salicina* in the Sangju area, Korea. The isolates KP1706, KP1729 and KP1736 were identified based on multi-locus (ITS, TUB2, GAPDH, ACT, and CHS-1) phylogenetic analysis and morphological characteristics.

***Colletotrichum nymphaeae*** (Pass.) Aa, Netherlands Journal of Plant Pathology, Supplement 1 84: 110. 1978.

For detailed illustrations of *C*. *nymphaeae* see Damm *et al*.^21^.

#### Materials examined

SOUTH KOREA, Gyeongbuk Province, Sangju City, from diseased fruit of *Prunus salicina*, 21 Jul. 2017, O. Hassan, Culture KP1707 and KP1722 (Fig. 4).

Notes: KP1707 and KP1722 in our study were confidently identified as *C*. *nymphaeae* based on multi-locus (ITS, TUB2, GAPDH, ACT, and CHS-1) phylogenetic analysis. Colony color, conidia (shape), and appressoria (shape) is comparable with some *Colletotrichum* species with in the *C*. *gloeosporioides* and *C. acutatum* species complex^20,21^. *Colletotrichum nymphaeae* separated clearly from other species based on multi - locus (ITS, TUB2, GAPDH, ACT, and CHS-1) molecular analysis, rather than morphological characteristics. *C*. *nymphaeae* was reported in our recent publication as the causative agent of plum anthracnose^9^.

### Pathogenicity assay

The pathogenicity of the *Colletotrichum* isolates was evaluated on detached plum fruits for confirmation of Koch’s postulates. All isolates of *Colletotrichum* showed anthracnose symptoms on plum fruit inoculated using the wounding approach, while only *C*. *siamense*, *C*. *nymphaeae* and *C*. *fioriniae* were capable of infecting non-wounded fruits as shown in Table 2. The *C*. *siamense* isolates produced the largest lesions on wounded fruits. *Colletotrichum siamense*, *C*. *nymphaeae* and *C*. *fioriniae* showed less virulence on non-wounded fruits in term of both disease incidence and lesion size.

**Table 2:** Pathogenicity testing of *Colletotrichum* species from Japanese Plum (*Prunus salicina*).

Species and isolates		Mean infection incidence (%)		Lesion diameter on fruits (mm)	
		Wounding	Non-wounding	Wounding	Non-wounding
*C. siamense*	KP1701	100	10	50.60 ± 3.30	9.3 ± 1.2
	KP1702	100	10	50.72 ± 3.50	8.0 ± 2.0
	KP1711	100	5	49.20 ± 3.92	6.3 ± 1.5
	KP1712	100	10	42.62 ± 1.50	7.3 ± 1.5
*C. gloeosporioides* s.s.	KP1705	100	0	20.60 ± 0.63	0
	KP1740	100	0	22.31 ± 0.35	0
*C. fioriniae*	KP1706	100	5	19.14 ± 2.33	4.7 ± 0.42
	KP1729	100	10	18.50 ± 1.01	4.6 ± 0.72
	KP1736	100	25	19.82 ± 1.23	5.12 ± 1.02
*C. nymphaeae*	KP1707	100	5	18.75 ± 1.70	3.25 ± 1.0
	KP1722	100	0	19.45 ± 1.36	0

## Discussion

Anthracnose and other diseases caused by *Colletotrichum* spp. on the leaves, stems and fruits of numerous important crops have become increasingly common in South Korea. The disease of anthracnose has severely limited commercial production of various important fruit crops, such as apples, peaches, persimmons, grapes, and others across South Korea^12–14,16,17^. Anthracnose on fruits causes severe losses because of both pre and post-harvest fruit decay, which makes the fruits completely unmarketable. Very recently, *Colletotrichum* anthracnose has been reported in Japanese plums in Korea^3,9^. In previous research, morphological and ITS sequence approaches has been used to identify *Colletotrichum* spp. responsible for anthracnose on Japanese plums^3^. Morphological characteristics along with ITS sequence analysis may be more beneficial for identifying isolates to species complex rather than specific species. In the present study, *Colletotrichum* species associated with plum anthracnose from Sangju, Korea were identified using a multilocus phylogenetic analysis approach followed by an evaluation of their pathogenicity. Four isolates were identified as *C*. *siamense*, two isolates as *C*. *gloeosporioides* s.s., three isolates as *C*. *fioriniae* and two isolates as *C*. *nymphaeae*.

*Colletotrichum siamense* is a member of *C*. *gloeosporioides* s.l. and is described here for the first time as responsible for plum anthracnose in Sangju, Korea. *C*. *gloeosporioides* s.s., and *C*. *nymphaeae* are species from the *C*. *gloeosporioides* species complex and the *C. acutatum* species complex respectively, that have been previously reported to cause anthracnose in plums in Korea^3.9^. *Colletotrichum fioriniae* was first reported as a species of *C. acutatum* s.l., to have cause plum anthracnose in Korea. Phylogenetic analysis and morphological data including colony characters and conidial measurements were previously used to distinguish four *Colletotrichum* species^20,21^. Morphological characteristics of *C*. *siamense* and *C*. *gloeosporioides* s.s., including colony characters, conidial measurements, and appressoria measurements overlapped with those of other species in *C*. *gloeosporioides* s.l. Identifying *Colletotrichum* species within *C*. *gloeosporioides* s.l., based on morphological characteristics is uncertain because of: (1) overlapping morphological characteristics among the species^20^ and (2) slight morphological differences that can be due to different growing conditions, temperature, light regime, and geographic isolates^20^. Multilocus (ITS, TUB2, GAPDH, ACT, CAL, and CHS) phylogeny analysis clearly showed that the present isolates, *C*. *siamense* and *C*. *gloeosporioides* s.s., clustered in a distinct phylogenetic clade with in *C*. *gloeosporioides* s.l., with a high posterior probability value (0.92) (Fig. 2). *C*. *siamense* isolates were further confirmed via both phylogenetic analysis and BLAST search using ApMat sequence data. ApMat is a potentially powerful gene disentangling both *C*. *gloeosporioides* s.l., and *C*. *siamense* complexes^22,24^. *Colletotrichum siamense* was previously considered as a species complex, but recent studies have shown *C. siamense* to be a single species based on molecular analyses using GCPSR as well as coalescent methods General Mixed Yule Coalescent and Poisson Tree Processes^23,24,28^. The PHI test result in the present study also found significant recombination among *C*. *siamense* species of different geographic origins. *Colletotrichum siamense* has been associated with anthracnose in various commercial crops^17,20,24^. To the best of our knowledge, this is the first report of anthracnose of plums caused *C*. *siamense* in Korea.

*C. gloeosporioides* s.s., is the most frequently reported plant pathogen causing anthracnose in a variety of hosts in Korea^6,30–33^. However, this is only the second report on plum anthracnose caused by *C. gloeosporioides* s.s. in Korea. It was previously identified based on morphological characteristics and the ITS sequence analysis, whereas here we identified it using multilogue phylogenetic analysis, which was supported by morphological characteristics evaluations. *Colletotrichum fioriniae* can be easily identified by the colony pigment on PDA, which is pink cottony with gray aerial mycelium in compact tufts from above and pink with flecking in reverse^35^. *Colletotrichum fioriniae* was previously reported as the causative agent of anthracnose on a variety of hosts^6,14,34^. To our knowledge, this is the first report of *C. fioriniae* causing anthracnose of plums in South Korea. *Colletotrichum nymphaeae* is reported as the causal agent of plum anthracnose for the second time here^9^.

The pathogenicity tests showed that the four species of *Colletotrichum* evaluated in this study are pathogenic to plum fruits and could be differentiated by the degree of virulence and lesion size in inoculated fruits. All *Colletotrichum* isolates tested caused anthracnose on wounded fruit, whereas only *C*. *siamense*, *C*. *nymphaeae* and *C*. *fioriniae* isolates were able to infect unwounded fruits. *C*. *siamense* isolates produced larger lesions on plum fruits followed by *C. gloeosporioides* s.s., *C*. *nymphaeae* and *C*. *fioriniae*. Koch’s postulates were fulfilled by re-isolating the fungus from the lesions of inoculated fruits and reidentifying them at the species level using morphological and multi-locus sequences approaches.

In conclusion, this study identified 2 species within *C. gloeosporioides* s.l., and 2 species within the *C. acutatum* complex. This investigation included only one area (Sangju) of Korea, which highlights the importance of further research on *Colletotrichum* strains isolated from different Korean regions to mitigate the risk to the plum fruit industry in Korea.

## Material and Methods

### Sample collection and isolation

Japanese plum fruits with visible anthracnose were collected in 2017 from different commercial orchards in Sangju Korea. The fruits were characterized by sunken, round, and brown necrotic lesions. Three diseased fruits were selected from each orchard for the isolation causal agents, and fruits were washed with distilled water. Causal agents were isolated from necrotic tissue of diseased fruits as follows. Small pieces (2 mm^2^) of necrotic tissue were removed aseptically with a scalpel, disinfected with a 1% NaOCl solution (w/v) for 1 min followed by three washes in sterile distilled water. After drying by blotting, the disinfected tissues were placed on water agar (WA) petri plates supplemented with streptomycin (0.05 g/L) and incubated at 25 °C in the dark. Newly emerging hyphae from the tissue were transferred onto fresh potato dextrose agar (PDA) petri plates and incubated at 25 °C in the dark. Pure fungal cultures were obtained using the single spore isolation technique from 7-day PDA cultures^18^. Conidial suspensions were prepared in sterile distilled water. The concentration of each conidial suspension was determined by using a hemocytometer. Then, the conidial suspensions (~10^4^ conidia/ml) were made from the concentrated suspensions. Conidial suspensions were then sprayed on to PDA plates and incubated in the dark at 25 °C. Single germinating spores were collected with a sterilized needle after overnight incubation and placed on fresh PDA plates and incubated in the dark at 25 °C. Seven-day-old cultures were grouped based on culture morphology and conidial shape.

### DNA extraction, PCR amplification, and sequencing

Fungal mycelia were acquired with a sterile scalpel from 4-day-old cultures of isolates grown on PDA, and total genomic DNA was extracted using a HiGeneTM Genomic DNA Prep Kit (Yuseong-Gu, Daejeon, Korea), following the manufacturer’s instructions. For *C. gloeosporioides* s.l. isolates, seven targeted genes were selected for PCR amplification and sequencing: internal transcribed spacer regions and intervening 5.8S nrRNA gene (ITS), glyceraldehyde-3-phosphate dehydrogenase (GAPDH), actin (ACT), beta-tubulin (TUB2), calmodulin (CAL), chitin synthase (CHS-1) and the Apn2–Mat1–2 intergenic spacer and partial mating type (Mat1–2) gene (ApMat). For *C. acutatum* s.l. isolates, five targeted genes were selected for PCR amplification and sequencing: ITS, GAPDH, ACT, TUB2, and CHS-1. The primer sets used in this study are listed in Table 3. The PCR amplifications were carried out in a simpliAmp™ thermal cycler (Thermo Fisher Scientific Inc). Each 25 μL PCR mixture consisted of 18.8 μL UV-sterilized ultra-filtered water, 2.5 µL 10x F-star *Taq* buffer, 0.5 µl dNTP Mix (each 10 mM), 1 µL forward primer (10 pmol), 1 µL reverse primer (10 pmol), 1 µL genomic DNA, and 0.2 µL F-star *Taq* DNA polymerase (BIOFACT, Korea). The PCR conditions were the same as the conditions applied for amplification of ITS using the universal primers ITS1F/ITS4, except for the annealing temperatures^20^. Locus-specific annealing temperatures are shown in Table 1. Purification and sequencing of the PCR product were performed commercially at Macrogen, Inc. (Seoul, Korea).

**Table 3:** Primers used in this study, including sequences and sources.

Gene	Primer Name	Direction	Sequence (5′-3′)	Annealing temperature (°C)	References
GAPDH	GDF	Forward	GCC GTC AAC GAC CCC TTC ATT GA	60	Templeton *et al*.^44^
	GDR	Reverse	GGG TGG AGT CGT ACT TGA GCA TGT	60	Templeton *et al*.^44^
ITS	ITS-1F	Forward	CTT GGT CAT TTA GAG GAA GTA A	55	Gardes & Bruns^45^
	ITS-4	Reverse	TCC TCC GCT TAT TGA TAT GC	55	White *et al*.^46^
CAL	CL1C	Forward	GAA TTC AAG GAG GCC TTC TC	59	Weir *et al*.^20^
	CL2C	Reverse	CTT CTG CAT CAT GAG CTG GAC	59	Weir *et al*.^20^
Actin	ACT-512F	Forward	ATG TGC AAG GCC GGT TTC GC	58	Carbone & Kohn^47^
	ACT-783R	Reverse	TAC GAG TCC TTC TGG CCC AT	58	Carbone & Kohn^47^
ApMat	AM-F	Forward	TCA TTC TAC GTA TGT GCC CG	62	Silva *et al*.^22^
	AM-R	Reverse	CCA GAA ATA CAC CGA ACT TGC	62	Silva *et al*.^22^
TUB2	Bt2a	Forward	GGT AAC CAA ATC GGT GCT GCT TTC	55	Glass & Donaldson^48^
	Bt2b	Reverse	ACC CTC AGT GTA GTG ACC CTT GGC	55	Glass & Donaldson^48^
CHS-1	CHS-79F	Forward	TGG GGC AAG GAT GCT TGG AAG AAG	58	Carbone & Kohn^47^
	CHS-345R	Reverse	TGG AAG AAC CAT CTG TGA GAG TTG	58	Carbone & Kohn^47^

### Phylogenetic analysis

The accession numbers for all sequences were acquired after depositing the resulting consensus sequences in GenBank (accession numbers are listed in Table 4). The generated sequences from the present isolates and those retrieved from GenBank (Table 4) for each gene were aligned using the MUSCLE multiple sequence alignment programs of MEGA v. 6.0^36^. Manually edited (if necessary) multiple sequence alignments were constructed for each gene, all gaps were treated as missing data and concatenated with Mesquite v. 2.75^37^. The phylogenetic analyses were performed using concatenated aligned sequences of different gene combinations. Neighbor-joining (NJ), maximum likelihood (ML), and maximum parsimony (MP) phylogenetic analyses were performed using MEGA v. 6.0^36^. Bayesian inference (BI) phylogenetic analyses were performed with MrBayes v. 3.2.2^38^. GTR + I + gamma mode determined using MrModeltest v. 2.3 was utilized to construct the Bayesian phylogenetic tree^39^. MCMC analysis of four chains based on the full dataset was run in parallel from a random tree topology, the heat parameter was set at 0.15, and trees were sampled every 100 generations. The MCMC analysis was stopped when the average standard deviation of split frequencies reached 0.01 (stop value). The first 25% of the generations were set as burn-in after which the likelihood values remained stationary. Consensus BI phylogenetic trees were viewed in FigTree v 1.3.1^40^. For the preliminarily identification of *Colletotrichum* isolates belonging to *C*. *gloeosporioides* s.l. and *C*. *acutatum* s.l., both the ITS and TUB2 alignment sequences were used for phylogenetic analysis. The sequences of five genes (ITS, TUB2, GAPDH, CHS-1, and ACT) were used for the phylogenetic analysis of isolates belonging to the *C*. *acutatum* species complex. The sequences of six genes (ITS, TUB2, GAPDH, ACT, CAL, and CHS-1) were used to analyze isolates belonging to *C*. *gloeosporioides* s.l. ApMat sequences were used for proper identification of *C*. *siamense* isolates.

**Table 4:** GenBank accession numbers of the *Colletotrichum* isolates used in this study for molecular data analyses.

Species	Isolate	GenBank accession number						
		GAPDH	ITS	ACT	CAL	ApMat	CHS-1	TUB2
*C. acerbum*	CBS 128530*	JQ948790	JQ948459	JQ949780	—	—	JQ949120	JQ950110
*C. acutatum*	CBS 112996*	JQ948677	JQ005776	JQ005839	—	—	JQ005797	JQ005860
	CBS 126521	JQ948697	JQ948366	JQ949687	—	—	JQ949027	JQ950017
*C. aenigma*	ICMP 18608*	JX010044	JX010244	JX009443	JX009683	KM360143	JX009774	JX010389
*C. aeschynomenes*	ICMP 17673*	JX009930	JX010176	JX009483	JX009721	KM360145	JX009799	JX010392
*C. alatae*	ICMP 17919*	JX009990	JX010190	JX009471	JX009738	KC888932	JX009837	JX010383
*C. alienum*	ICMP 12071*	JX010028	JX010251	JX009572	JX009654	KM360144	JX009853	JX010411
*C. aotearoa*	ICMP 18532	JX010005	JX010205	JX009564	JX009611	KC888930	JX009882	JX010420
*C. asianum*	ICMP 18580*	JX009915	FJ972612	JX010053	FJ917506	FR718814	JX009867	JX010406
*C. australe*	CBS 116478*	JQ948786	JQ948455	JQ949776	—	—	JQ949116	JQ950106
*C. boninense*	MAFF305972*	HM585386	HM585399	HM582001	HM582004	—	—	HM585399
*C. brisbanense*	CBS 292.67*	JQ948603	JQ948273	JQ949594	—	—	JQ948934	JQ949924
*C. chrysanthemi*	IMI 364540	JQ948601	JQ948271	JQ949592	—	—	JQ948932	JQ949922
*C. clidemiae*	ICMP 18658*	JX009989	JX010265	JX009537	JX009645	KC888929	JX009877	JX010438
*C. cordylinicola*	ICMP 18579	JX009975	JX010226	HM470235	HM470238	JQ899274	JX009864	JX010440
*C. cosmi*	CBS 853.738*	JQ948604	JQ948274	JQ949595	—	—	JQ948935	JQ949925
*C. costaricense*	CBS 330.75*	JQ948510	JQ948180	JQ949501	—	—	JQ948841	JQ949831
*C. cuscutae*	IMI 304802*	JQ948195	JQ948525	JQ949516	—	—	JQ948856	JQ949846
*C. fioriniae*	CBS 125396	JQ948629	JQ948299	JQ949620	—	—	JQ948960	JQ949950
	CBS125396	JQ948655	JQ948325	JQ949646	—	—	JQ948986	JQ949976
	KP17*06*	LC406922	LC406908	LC406929	—	—	LC406942	LC406915
	KP1729	LC438773	LC438765	LC438777	—	—	LC438781	LC438769
	KP1736	LC438774	LC438766	LC438778	—	—	LC438782	LC438770
*C. fructicola*	ICMP 18581*	JX010033	JX010165	FJ907426	FJ917508	JQ807838	JX009866	JX010405
*C. gloeosporioides* s.s.	ICMP 17821*	JX010056	JX010152	JX009531	JX009731	JQ807843	JX009818	JX010445
	KP1705	LC406920	LC406906	LC406927	LC406934	LC406940	LC438787	LC406913
	KP1740	LC406921	LC406907	LC406928	LC406935	LC406941	LC438788	LC406914
*C. godetiae*	CBS 133.44*	JQ948733	JQ948402	JQ949723	—	—	JQ949063	JQ950053
*C. guajavae*	IMI 350839*	JQ948600	JQ948270	JQ949591	—	—	JQ948931	JQ949921
*C*. *horii*	NBRC 7478*	GQ329681	GQ329690	JX009438	JX009604	JQ807840	JX009752	JX010450
*C. indonesiense*	CBS 127551*	JQ948618	JQ948288	JQ949609	—	—	JQ948949	JQ949939
*C. johnstonii*	CBS 128532*	JQ948775	JQ948444	JQ949765	—	—	JQ949105	JQ950095
*C. kahawae*	ICMP 18539*	JX009966	JX010230	JX009523	JX009635	JQ894579	JX009813	JX010434
*C. kinghornii*	CBS 198.35*	JQ948785	JQ948454	JQ949775	—	—	JQ949115	JQ950105
*C. laticiphilum*	CBS 1129898	JQ948619	JQ948289	JQ949610	—	—	JQ948950	JQ949940
*C.limetticola*	CBS 114.14*	JQ948523	JQ948193	JQ949514	—	—	JQ948854	JQ949844
*C. lupini*	CBS 109225*	JQ948485	JQ948155	JQ948816	—	—	JQ949476	JQ949806
*C. melonis*	CBS 159.84*	JQ948524	JQ948194	JQ949515	—	—	JQ948855	JQ949845
*C. musae*	CBS 116870*	JX010050	JX010146	JX009433	JX009742	KC888926	JX009896	HQ596280
*C. nupharicola*	CBS 480.96*	JX009972	JX010187	JX009437	JX009663	JX145319	JX009835	JX010398
*C. nymphaeae*	CBS 100065	JQ948555	JQ948225	JQ949546	—	—	JQ948886	JQ949876
	KP1707	LC438771	LC438763	LC438775	—	—	LC438779	LC438767
	KP1722	LC438772	LC438764	LC438776	—	—	LC438780	LC438768
*C. orchidophilum*	CBS 632.80*	JQ948481	JQ949472	JQ948524	—	—	JQ948512	JQ949802
*C. paxtonii*	IMI 165753*	JQ948615	JQ948285	JQ949606	—	—	JQ948946	JQ949936
*C. phormii*	CBS 118194*	JQ948777	JQ948446	JQ949767			JQ949107	JQ950097
*C. pseudoacutatum*	CBS 436.77*	JQ948811	JQ949801	JQ948777	—	—	JQ949141	JQ950131
*C. pyricola*	CBS 128531*	JQ948776	JQ948445	JQ949766	—	—	JQ949106	JQ950096
*C. psidii*	CBS 145. 29 *	JX009967	JX010219	JX009515	JX009743	KC888931	JX009901	JX010443
*C. rhombiforme_*	CBS 129953*	JQ948788	JQ948457	JQ949778	—	—	JQ949118	JQ950108
*C. queenslandicum*	ICMP1778*	JX009934	JX010276	JX009447	JX009691	KC888928	JX009899	JX010414
*C. salicis*	CBS 607.94	JQ948791	JQ948460	JQ949781	—	—	JQ949121	JQ950111
*C. salsolae*	ICMP 19051*	JX009916	JX010242	JX009562	JX009696	KC888925	JX009863	JX010403
*C. scovillei*	CBS 126529*	JQ948597	JQ948267	JQ949588	—	—	JQ948928	JQ949918
*C*. *siamense*	ICMP 18578*	JX009924	JX010171	FJ907423	FJ917505	JQ899289	JX009865	JX010404
*C*. *siamense* (syn. *C*. *hymenocallidis)*	ICMP18642	JX010019	JX010278	GQ856775	JX009709	JQ899283	GQ856730	JX010410
*C*. *siamense*	KP1701	LC406916	LC406902	LC406923	LC406930	LC406936	LC438783	LC406909
	KP1702	LC406917	LC406903	LC406924	LC406931	LC406937	LC438784	LC406910
	KP1711	LC406918	LC406904	LC406925	LC406932	LC406938	LC438785	LC406911
	KP1712	LC406919	LC406905	LC406926	LC406933	LC406939	LC438786	LC406912
*C. theobromicola*	CBS124945	JX010006	JX010294	JX009444	JX009591	KC790726	JX009869	JX010447
*C. ti*	ICMP 4832	JX009952	JX010269	JX009520	JX009649	KM360146	JX0010123	JX010442
*C. tropicale*	ICMP 18653*	JX010007	JX010264	JX009489	JX009719	KC790728	JX010097	JX010407
*C. walleri*	CBS 125472*	JQ948605	JQ948275	JQ949596	—	—	JQ948936	JQ949926
*C. xanthorrhoeae*	BRIP 45094*	JX009927	JX010261.	JX009478	JX009653	KC790689	JX009823	JX010448

*Ex-holotype or ex-epitype cultures.

### Genealogical concordance phylogenetic species recognition analysis

The Genealogical Concordance Phylogenetic Species Recognition (GCPSR) model was used to analyze the phylogenetically related, but ambiguous species as described by Quaedvlieg *et al*. by performing a pairwise homoplasy index (Φw, PHI) test^41^. The PHI test was performed in Splits Tree 4^42,43^. A six-locus concatenated dataset (ITS, TUB2, GAPDH, ACT, CAL, and CHS-1) of closely related species (Fig. 2) and a five-locus concatenated dataset (ITS, TUB2, GAPDH, ACT, and CHS-1) of closely related species (Fig. 3) were used to determine the recombination level and both the LogDet transformation and splits decomposition options were selected^41^. The PHI test value below a 0.05 threshold (Φw < 0.05) indicated significant recombination in the dataset.

### Morphological characterization

All selected isolates were described based on culture morphology and growth rate, and conidia as well as appressoria shape and size. Cultures were grown on PDA using mycelial discs (5 mm diameter) from 5-day-old cultures at 25 °C under 16 h light/8 h dark conditions. Culture diameter was measured each day, and the appearance was evaluated after 7 days of growth. The daily growth rate was calculated based on measurement from six replicates. Conidial characteristics (size and shape) were determined using conidia taken from the conidial mass on the culture and mounted on glass slides in clear lactic acid; the length and wide of 50 conidia were measured for each isolate. For appressoria production, conidia mounted on glass slides in distilled water were placed in Petri dishes containing a moistened tissue and incubated at 25 °C under 16 h light/8 h dark conditions. After two days of incubation, appressoria that formed across the underside of the coverslip were measured; the size of 50 appressoria was measured for each isolate. Conidia and appressoria sizes were measured with a stage micrometer under an Olympus BX43 microscope (Olympus Corporation, Japan) at 400× magnification.

### Pathogenicity tests

All eleven isolates were subjected to pathogenicity tests on Japanese plum. Mature detached Japanese plum fruits were collected from Sangju Emart and used for the pathogenicity assay. The collected plum fruits were washed with tap water and then disinfected for 3 minutes in 1% sodium hypochlorite, followed by washing with sterile distilled water three times. Disinfected fruits were placed in a plastic container and inoculated with conidial suspension of the respective isolates using both nonwounding and wounding methods. A 10^6^ conidia/mL conidial suspension was made from 7-day-old cultures of each isolate, as described above. For the wounding method, fruits were wounded by pricking with a sterile needle and a 10 µL droplet of the conidial suspension was placed at the wounded point. For the non-wounding method, the conidial suspension was sprayed over the fruits surface until surface runoff was observed. Control fruits for both methods received distilled water. Ten fruits were used for each treatment. After inoculation the plastic containers were sealed and incubated at 25 °C in the dark under high humidity conditions in an incubator. After 5 days of incubation, anthracnose lesions were observed on fruits inoculated with fungal conidia. Control fruits remained symptom-free. The disease incidence (DI) was expressed as the percentage of infected fruits compared to the total number of inoculated fruits. A ruler was used to measure lesion diameters (LDs). Causal agents were isolated from infected fruits, cultured on a new PDA plate, and then identified according to the methods described above to confirm Koch’s postulates.

### Statistical analysis

MS Excel was used to calculate the average and standard deviation of each data sets. Values for daily growth rate, conidia and appressor sizes, and lesion diameters expressed as the average ± standard deviation (av ± SD).

### Comment

The photograph(s) in figure 4 were obtained from Sangju, Korea, and the images were taken by Oliul Hassan (O.H) and Taehyun Chang (T.C).

## Supplementary information

Supplementary information
