# Supplementary material for: Colletotrichum Species Associated with Japanese Plum (Prunus salicina) Anthracnose in South Korea
Source: Sci Rep. 2019 Aug 19;9:12089. doi: 10.1038/s41598-019-48108-1 (PMC6700192; doi:10.1038/s41598-019-48108-1)
Supplement: Supplementary file 1 — Supplementary information [file 41598_2019_48108_MOESM1_ESM.pdf]

Colletotrichum Species Associated with Japanese Plum (*Prunus salicina*) Anthracnose in South Korea

Oliul Hassan[1], Yong Se Lee[2] and Taehyun Chang[1*]

[1] Department of Ecology & Environmental System, College of Ecology & Environmental Sciences, Kyungpook National University, Sangju, Gyeongbuk 37224, Korea (Republic of)

[2] Division of Life and Environmental Sciences, College of Life and Environmental Sciences, Daegu University, Gyeongsan, Gyeongbuk 38453, Korea (Republic of)

***Corresponding Author**: Tel: +82 54 530 1204, Fax: +82-54-530-1209, E-mail: thchang@knu.ac.kr

Appendices legends

Appendix 1: Maximum likely hood tree based on AptMat sequence alignment.

Appendix 2: The results of the pairwise homoplasy index (PHI) test of closely related species using both LogDet transformation and splits decomposition. PHI test results (Φw) < 0.05 indicate significant recombination within the dataset.

Appendix 1:

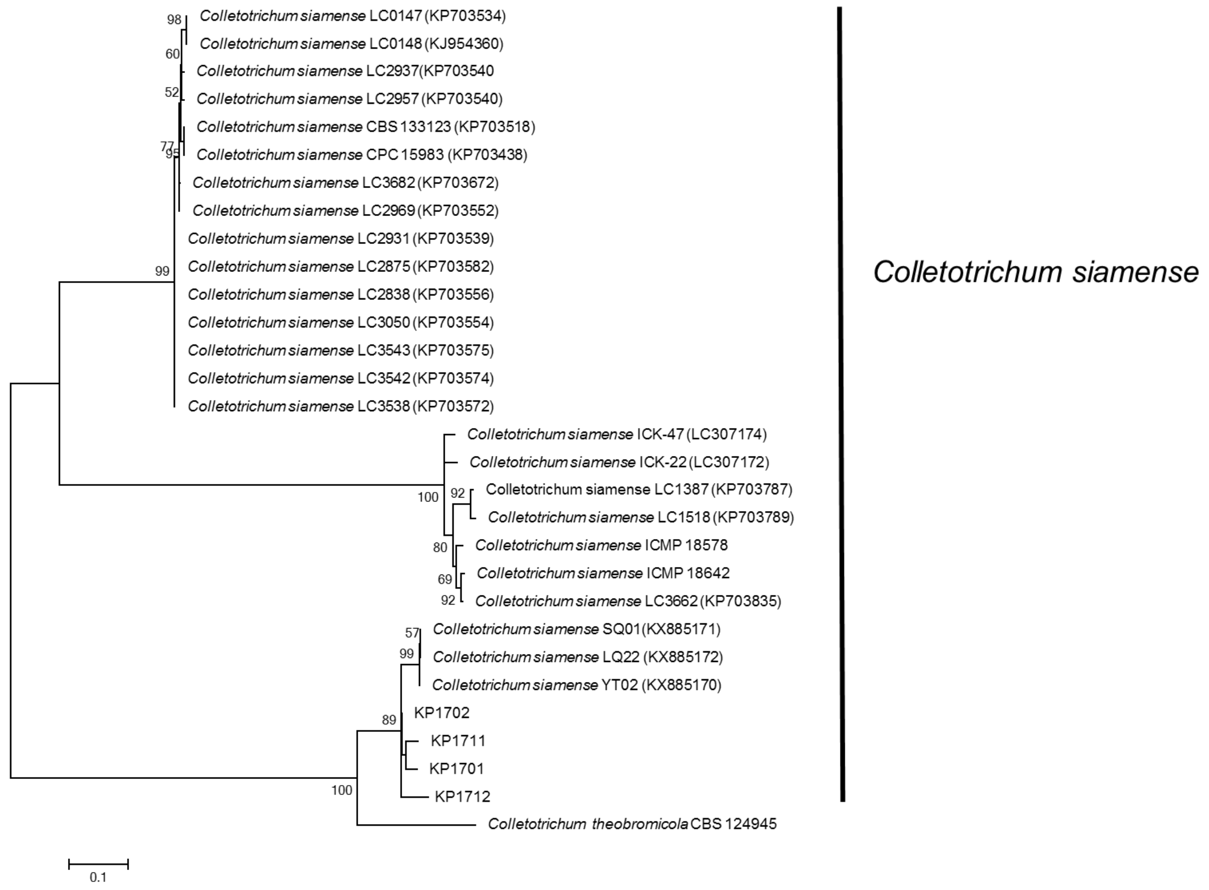
Colletotrichum siamense LC0147 (KP703534)
Colletotrichum siamense LC0148 (KJ954360)
Colletotrichum siamense LC2937(KP703540
Colletotrichum siamense LC2957 (KP703540)
Colletotrichum siamense CBS 133123 (KP703518)
Colletotrichum siamense CPC 15983 (KP703438)
Colletotrichum siamense LC3682 (KP703672)
Colletotrichum siamense LC2969 (KP703552)
Colletotrichum siamense LC2931 (KP703539)
Colletotrichum siamense LC2875 (KP703582)
Colletotrichum siamense LC2838 (KP703556)
Colletotrichum siamense LC3050 (KP703554)
Colletotrichum siamense LC3543 (KP703575)
Colletotrichum siamense LC3542 (KP703574)
Colletotrichum siamense LC3538 (KP703572)
Colletotrichum siamense ICK-47 (LC307174)
Colletotrichum siamense ICK-22 (LC307172)
Colletotrichum siamense LC1387 (KP703787)
Colletotrichum siamense LC1518 (KP703789)
Colletotrichum siamense ICMP 18578
Colletotrichum siamense ICMP 18642
Colletotrichum siamense LC3662 (KP703835)
Colletotrichum siamense SQ01(KX885171)
Colletotrichum siamense LQ22 (KX885172)
Colletotrichum siamense YT02 (KX885170)
KP1702
KP1711
KP1701
KP1712
Colletotrichum theobromicola CBS 124945
Colletotrichum siamense
98
60
52
77
95
99
100
92
80
69
92
57
99
89
100
0.1

Appendix -2:

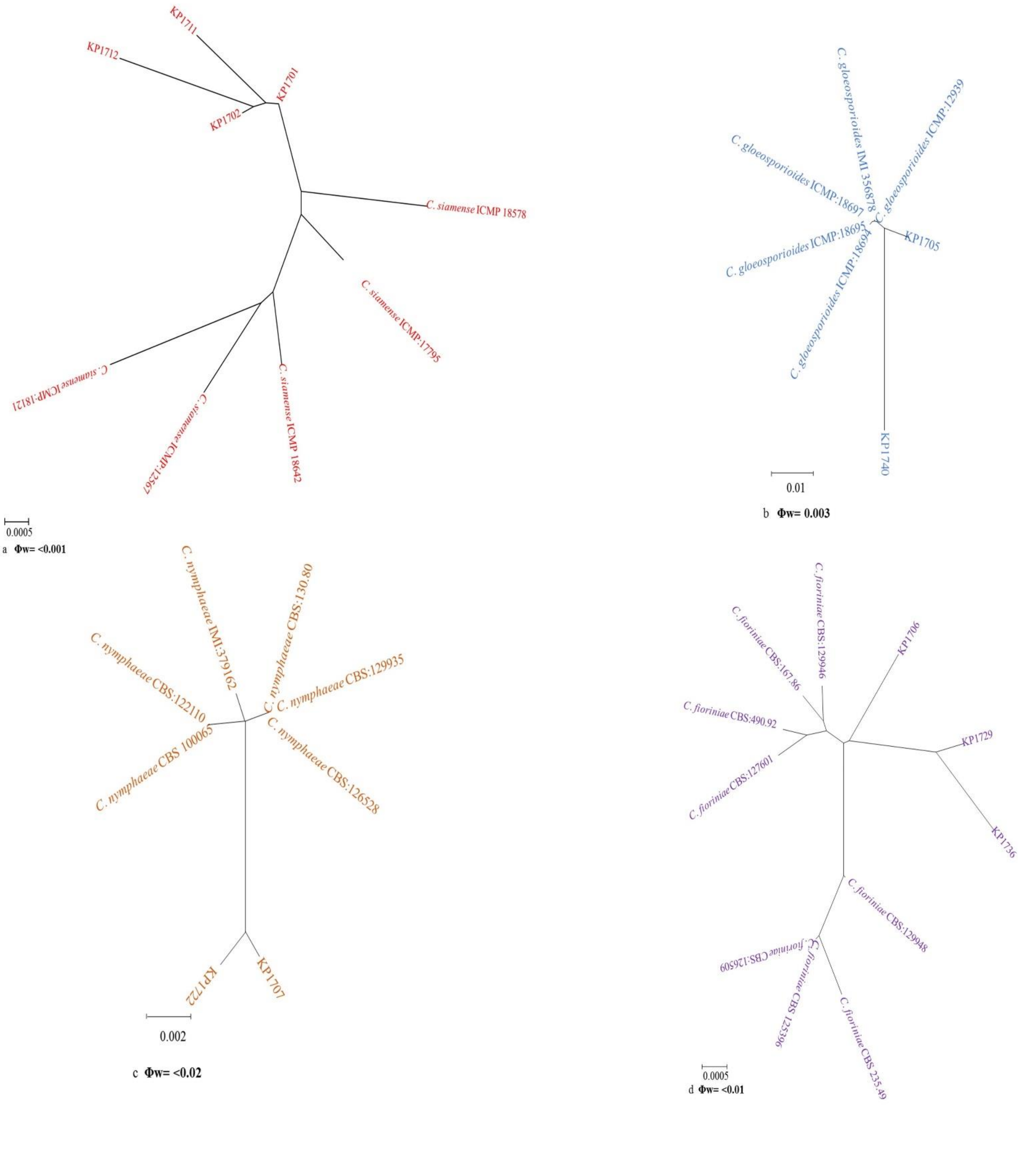
KP1711
KP1712
KP1701
KP1702
C. siamense ICMP 18578
C. siamense ICMP:17795
C. siamense ICMP:18121
C. siamense ICMP:12567
C. siamense ICMP 18642
0.0005
a Φw= <0.001
C. gloeosporioides IMI 356878
C. gloeosporioides ICMP:12939
C. gloeosporioides ICMP:18697
C. gloeosporioides ICMP:18695
C. gloeosporioides ICMP:18694
KP1705
KP1740
0.01
b Φw= 0.003
C. nymphaeae IMI:379162
C. nymphaeae CBS:130.80
C. nymphaeae CBS:122110
C. nymphaeae CBS:129935
C. nymphaeae CBS:126528
C. nymphaeae CBS 100065
KP1722
KP1707
0.002
c Φw= <0.02
C. fioriniae CBS:129946
C. fioriniae CBS:167.86
KP1706
C. fioriniae CBS:490.92
KP1729
C. fioriniae CBS:127601
KP1736
C. fioriniae CBS:129948
C. fioriniae CBS:126509
C. fioriniae CBS 125396
C. fioriniae CBS 235.49
0.0005
d Φw= <0.01
